# The use of induced hypothermia in extracorporeal membrane oxygenation: A narrative review

**DOI:** 10.1016/j.resplu.2023.100360

**Published:** 2023-01-28

**Authors:** Anthony Moreau, Bruno Levy, Filippo Annoni, Roberto Lorusso, Fuhong Su, Mirko Belliato, Fabio Silvio Taccone

**Affiliations:** a Department of Intensive Care, Hôpital Universitaire de Bruxelles (HUB), Brussels, Belgium; b Laboratoire Expérimental des Soins Intensifs, Université Libre de Bruxelles (ULB), Brussels, Belgium; c Service de Médecine Intensive et Réanimation Brabois, CHRU Nancy, Pôle Cardio-Médico-Chirurgical, Vandoeuvre-les-Nancy, France; d INSERM U1116, Faculté de Médecine, Université de Lorraine, 54000 Nancy, France; e Department of Cardio-Thoracic Surgery, Heart & Vascular Centre, Maastricht University Medical Centre, Cardiovascular Research Institute Maastricht (CARIM), Maastricht, The Netherlands; f UOC AR 2-Anestesia e Rianimazione Cardiotoracica Foundation IRCCS Policlinico San Matteo, Pavia, Italy

**Keywords:** Brain, Monitoring, ECMO, Ischemia, Perfusion, Induced hypothermia, Targeted temperature management, Cardiogenic shock, Acute distress respiratory syndrome, Cardiac arrest

## Abstract

Despite venovenous or venoarterial extracorporeal membrane oxygenation (ECMO) being increasingly used in patients with severe acute respiratory disease syndrome, severe cardiogenic shock, and refractory cardiac arrest, mortality rates still remain high mainly because of the severity of the underlying disease and the numerous complications associated with initiation of ECMO. Induced hypothermia might minimize several pathological pathways present in patients requiring ECMO; even though numerous studies conducted in the experimental setting have reported promising results, there are currently no recommendations suggesting the routine use of this therapy in patients requiring ECMO. In this review, we summarized the existing evidence on the use of induced hypothermia in patients requiring ECMO. Induced hypothermia was a feasible and relatively safe intervention in this setting; however, the effects on clinical outcomes remain uncertain. Whether controlled normothermia has an impact on these patients compared with no temperature control remains unknown. Further randomized controlled trials are required to better understand the role and impact of such therapy in patients requiring ECMO according to the underlying disease.

## Introduction

Extracorporeal membrane oxygenation (ECMO) therapy has been increasingly implemented in the management of critically ill patients worldwide. Despite the lack of strong evidence, venovenous (V-V ECMO) configuration has become a valuable therapy for severe acute respiratory distress syndrome (ARDS), which remains refractory to all recommended interventions, including protective ventilation, titration of positive end-expiratory pressure (PEEP), and prone position,^1^ while venoarterial (V-A ECMO) mode has been implemented for cardiogenic shock (CS) and refractory cardiac arrest [i.e., extracorporeal cardiopulmonary resuscitation (ECPR)].^2^ Nevertheless, mortality rates in those populations remain high^3^, ^4^, ^5^ and there is an urgent need to better understand how to improve the performance of ECMO devices to minimize complications and potentially provide organ protection.

Targeted temperature management (TTM) encompasses all the interventions required to reach and maintain a specific level of body temperature in critically ill patients. In particular, TTM is a common intervention in the management of brain-injured patients, following an anoxic or a traumatic injury.^6^, ^7^ Within different TTM strategies, the interest in induced hypothermia (IH), i.e., cooling the patient below normal body temperature (<37 °C), has always been very high because of its potential protective effects not only on the brain but also on other organs,^8^ at least as suggested in experimental studies. Indeed, minutes or hours after an acute injury (i.e., ischemia, inflammation, trauma), destructive processes begin at the cellular level, including cellular apoptosis, mitochondrial dysfunction, excessive free radical production, reperfusion injury, increased permeability of the blood–brain barrier and of the cellular membranes, accumulation of excitatory neurotransmitters, production of pro-inflammatory cytokines, and microthrombi formation.^8^, ^9^, ^10^ These processes may continue hours or days after the initial trigger, and most of those are temperature-dependent, i.e., they are enhanced by increasing body temperature.^8^ In this setting, IH can diminish the systemic and cerebral metabolism, resulting in a reduction in glucose and oxygen consumption, which would minimize the cellular metabolic distress.^8^, ^11^ IH can also decrease the carbon dioxide production, which would decrease the risk of intracranial and pulmonary hypertension.^8^ By inhibiting the caspase activation,^12^ IH would also prevent mitochondrial dysfunction and interrupt the apoptotic pathway, thus preventing cellular death.^13^ Finally, IH can attenuate the neuroexcitatory cascade, which occurs after cerebral ischaemia–reperfusion,^14^ reduce the local neutrophil and macrophage activation, which would sustain neuroinflammation,^8^, ^15^ and limit the production of free radicals.^16^ In addition to these cellular effects, IH can also affect the cardiovascular system [i.e., elevation of mean arterial pressure and stroke volume (SV)].^17^

In this review, we summarized the existing evidence on the use of TTM [i.e., including IH or controlled normothermia (NT)] in adult patients requiring ECMO. The PubMed database was searched from 1990 to 10 December 2022 using the keywords “((ECMO) OR (extracorporeal membrane oxygenation) OR (ECPR) OR (extracorporeal cardiopulmonary resuscitation) OR (ECLS) OR (extracorporeal life support)) AND ((hypothermia) OR (target temperature management) OR (TTM)). Moreover, the following MESH terms were also used – (ECMO) AND (hypothermia, induced)” – to identify the most relevant publication in this field. Only peer-reviewed published studies (case reports, retrospective/prospective studies, randomized controlled studies, and meta-analyses) written in English were considered to prepare this article.

## The rationale for induced hypothermia during ECMO

The application of TTM in patients requiring V-V or V-A ECMO is supported by different pathophysiological considerations. In these two situations, initiation of ECMO is associated with a complex inflammatory reaction characterized by the activation of coagulative and inflammatory cascades, the production of proinflammatory cytokines, the activation of the complement, and the innate immune system.^18^, ^19^ If severe and persistent, this inflammatory response may lead to endothelial injury, disrupted microcirculation, and organ dysfunction. In some clinical settings (i.e., ARDS, CS), an overwhelming inflammation is also frequently reported. Because of its anti-inflammatory properties, TTM may be then beneficial in these conditions.^20^, ^21^, ^22^, ^23^

In patients with refractory and persistent hypoxemia despite V-V ECMO, some strategies are currently available. The main determinant of oxygen saturation (SaO_2_) in patients undergoing V-V ECMO is the ratio of ECMO blood flow to cardiac output (Q_ECMO_/Q_CO_). In particular, a Q_ECMO_/Q_CO_ ratio of at least 0.6 was associated with a SaO_2_ value greater than 90%.^24^ Indeed, arterial oxygen content can be improved either by increasing ECMO flow or increasing blood oxygen delivery (i.e., red blood cell transfusion).^25^, ^26^ Recirculation, a phenomenon that occurs when the arterial cannula and the veinous cannula are too close or in case of very high ECMO blood flow, must also be minimized. Optimalization of mechanical ventilation (i.e., PEEP titration, alveolar recruitment maneuvers) or reduction in the intrapulmonary shunt (i.e., prone position, inhaled nitric oxide) may also be considered. If the patient remains hypoxemic despite all these interventions, a reduction in Q_CO_ could be considered using beta-blocker agents or increasing sedatives. Although experimental, IH is a practical option to reduce Q_CO_ and therefore increase Q_ECMO_/Q_CO_ ratio.^27^ Indeed, basal metabolic rate decreases by 6–10% for each degree of reduction in body temperature below 37 °C^8^, ^28^; as such, oxygen consumption and carbon dioxide production will also decrease by a similar proportion, resulting in higher arterial oxygen pressure (PaO_2_) and decreased arterial carbon dioxide pressure (PaCO_2_), without changes in the ventilator or ECMO settings. In particular, tissues and organs with a high baseline oxygen consumption, similar to the brain or the heart, have a proportionally greater reduction in oxygen consumption. Interestingly, reduction in carbon dioxide production may favor an even further reduction in minute ventilation (i.e., tidal volume and respiratory rate), reducing the respiratory mechanical power^29^ and then preventing the risk of ventilator-induced lung injury. Moreover, IH can enhance fat metabolism, leading to increased levels of glycerol, free fatty acids, ketonic acids, and lactate, resulting in mild metabolic acidosis^8^; through the Bohr effect, acidemia then induces a reduced hemoglobin affinity for oxygen and high oxygen tissue availability.^25^, ^30^

CS is the most severe form of acute heart failure and is frequently associated with an increased risk of death.^31^ There are numerous causes of CS, but the pathophysiology comprises several unique overlapping components^31^: an initial cardiac insult decreasing cardiac output (CO), central hemodynamic alterations, microcirculatory dysfunction, systemic inflammatory response syndrome, and multiorgan dysfunction. Central hemodynamic alterations include a reduction of stroke volume (SV), reduction in systemic arterial blood pressure (ABP), and elevation of left ventricular end-diastolic pressure.^31^ In this setting, IH may result in increased systemic vascular resistances^19^ and could potentially stabilize the patients with CS, although the reduction in heart rate would result in a slight reduction in CO. The use of IH in these patients may also help limit the severity of the ischemia–reperfusion injury.^31^ Importantly, V-A ECMO could be associated with some complications, such as an increased afterload (i.e., retrograde arterial flow), an increased myocardial work (i.e., more extensive ischemic injury), and an important inflammatory response (i.e., because of the blood contact with a large surface of the extracorporeal circuits).^32^ The use of IH might mitigate all these phenomena related to ECMO implementation.

**Fig. 1:**
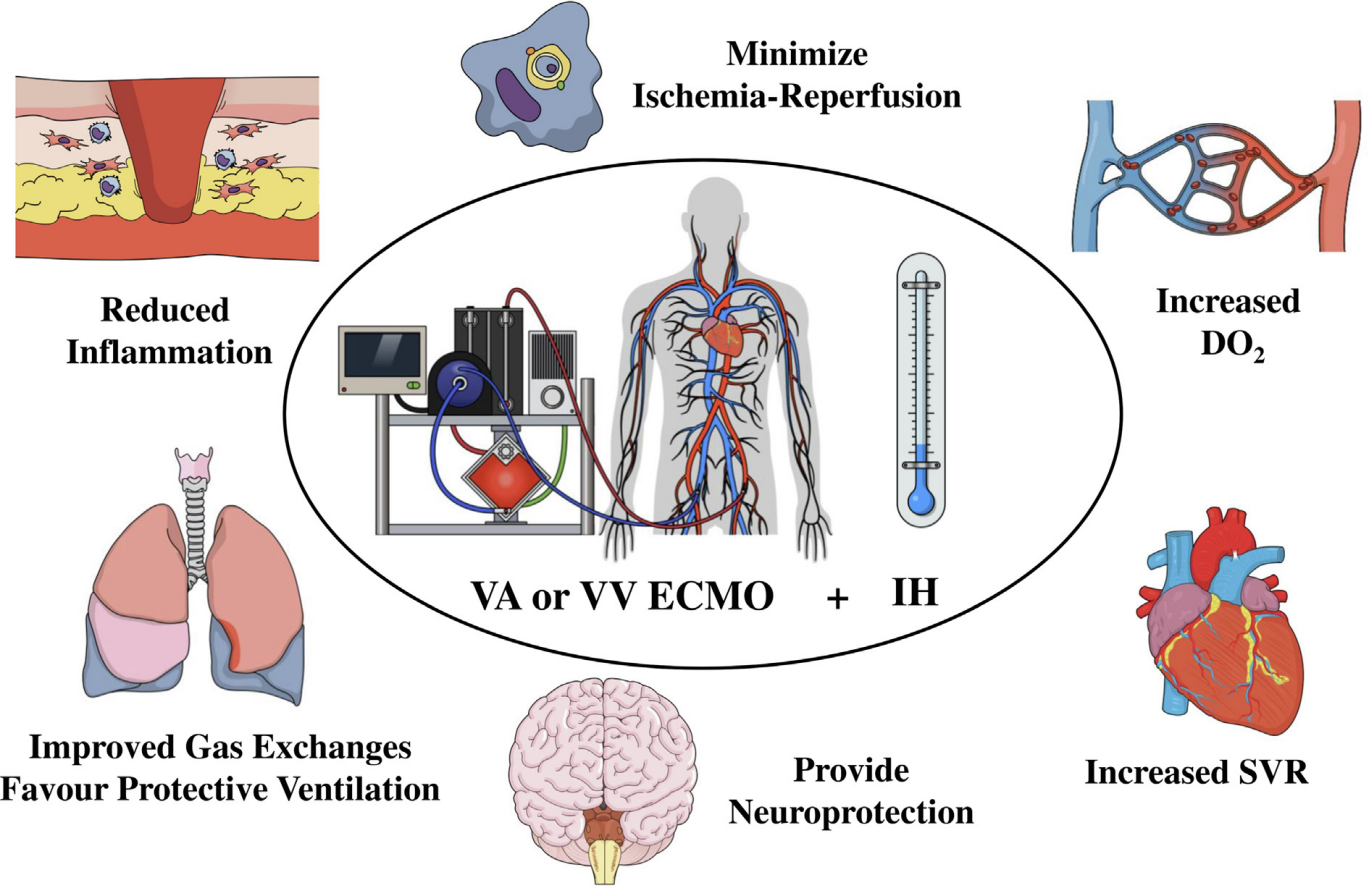
Potential benefits from induced hypothermia (IH) in patients with venoarterial (V-A) or venovenous (V-V) extracorporeal membrane oxygenation (ECMO) therapy.

Finally, IH may be interesting to improve neurological outcome in patients with refractory CA requiring ECPR. Indeed, these patients are exposed to a prolonged cardiopulmonary resuscitation and therefore are more susceptible to develop hypoxic ischemic brain injuries (HIBI). Pathophysiology of HIBI is complex and could be described as a two-hit model; the primary lesion is secondary to the cessation of oxygen transport during CA and the secondary lesion because of apoptosis, activation of a neuroexcitatory cascade with an excessive production of glutamate, and an inflammatory response. By limiting or minimizing all these mechanisms, IH could therefore be neuroprotective in these patients (Fig. 1).

## Induced hypothermia in animal models of ECMO

Few studies evaluating the use of TTM in animal models with ECMO have been published so far; all of them have evaluated the use of IH. Moreover, with the exception of one experiment realized in the setting of CS,^40^ all other studies were conducted in CA models.^33^, ^34^, ^35^, ^36^, ^37^, ^38^, ^39^

Table 1 summarizes the studies conducted on animal models. These studies showed several benefits from IH compared with the NT group. Regardless of the animal species, six studies demonstrated positive effects of IH on brain physiology by either resulting in better neurological outcomes (evaluated by neurological scores such as Neurologic Deficit Score or Overall Performance Category),^33^, ^34^, ^38^ reducing neuronal deaths (assessed by histology and molecular biology),^33^, ^36^, ^39^ or improving cerebral oxygenation.^36^ IH was also associated with reduced myocardial necrosis on histological and blood samples^33^, ^35^ and improved haemodynamics.^35^ Survival time was also longer in the IH group compared with normothermia.^34^, ^38^

**Table 1:** Summary of experimental data evaluating the use of induced hypothermia (IH) during the use of extracorporeal membrane oxygenation (ECMO).

Studies	Species	N	Study protocol	TTM protocol applied	Main outcomes
Ao et al., 2001^33^	Dog	17	Induction of VF by electric shock No Flow: 15 min ECPR during 24 h ICU stay during 72 h	*n* = 8: 33 °C for 20 h, then 37.5 °C until the end of the study *n* = 9: 37.5 °C throughout the study Core temperature reached 34 °C within the 30 min of the start of the cooling	NDS significantly lower in IH group Less degenerating pyramidal hippocampal neurons at brain biopsy in IH group Myocardial necrosis significantly lower in IH group
Han et al., 2010^34^	Rodent	35	CA by asphyxia (8 min) ECPR during 1 h ICU support during 1 h TTM during additional 6 h Observation during additional 3 days	*n* = 10: 37 °C *n* = 10: 34 °C *n* = 10: 30 °C *n* = 5: Sham No information about time to target temperature	72 h survival significantly higher in the 2 groups with IH than in NT group (80% in mild IH group vs 60% in moderate IH group vs 0% in NT group) NDS better at 24 h post resuscitation in the moderate IH group compared to the other groups OPC better in moderate IH group at 72 h post resuscitation than the two other groups
Ostadal et al., 2013^35^	Pig	8	Induction of VF by electric shock No Flow: 20 min ECPR during 90 min ICU stay during 72 h	*n* = 4: 33 °C during ECPR *n* = 4: 36.8 °C during ECPR 33 °C within the 5 min of the start of the cooling	ABP and cerebral oxygenation values significantly higher in IH group Levels of troponin I, myoglobin, CPK, and ALT significantly lower in the IH group Levels of NSE and CysC comparable
Janata et al., 2013^36^	Rodent	70	Induction of VF by electric shock No flow: 6 min ECPR groups: ECPR weaned from ECMO 2 min after ROSC	*n* = 13: ECPR + NT (37 °C for 12 h after ROSC) *n* = 13: ECPR + IH (33 °C for 12 h after ROSC) *n* = 13: CPR + NT (37 °C for 12 h after ROSC) *n* = 21: CPR + IH (33 °C for 12 h after ROSC) *n* = 10: sham 33 °C within the 10 min of the start of the cooling	NDS, OPC and surviving neurons in hippocampus were not statically different at day 14 between the 4 groups Significant reduction of neuronal death in subiculum and thalamus in IH groups No synergistic benefit from the combination of ECPR and IH
Bergan et al., 2016^37^	Pig	20	Induction of VF by electric shock No Flow: 15 min ECPR during 150 min	*n* = 10: 38 °C during 150′ *n* = 10: 32–33 °C during 120′ followed by 30′ of rewarming 33 °C within the 20 min of the start of the cooling	No effect of two-hour IH on resuscitation success No effect of two-hour IH on post-arrest left ventricular function (evaluated by LV pressure measurements and MRI) No effect of two-hour IH on magnitude of myocardial injury (estimated by serum concentrations of Troponin T and AST)
Foerster et al., 2018^38^	Pig	21	Induction of VF by electric shock No Flow: 20 min ECPR during 60 min	*n* = 11: 37 °C during 60′ *n* = 10: 32 °C during 30′ followed by 30′ of rewarming to a target of 36 °C 32 °C within the 15 min of the start of the cooling	Seven-day mortality, NDS at 24 h and at 120 h after CA significantly lower in the IH group Significantly less pyrexia or seizures at 24 h in IH group NDS on day 7 lower (NS) in the IH group
Zhang et al., 2021^39^	Pig	18	Induction of VF by electric shock No Flow: 6 min ECPR till 32 h post ROSC	*n* = 6: 37 °C during 32 h *n* = 6: 34 °C during 24 h followed by 8 h of rewarming (rewarming rate ≤ 0.5 °C/h) *n* = 6: 33 °C for 1 hour, alternate 35 °C for another hour during 24 h followed by 8 h of rewarming (rewarming rate ≤ 0.5 °C/h) IH within 2 h post ROSC	IH significantly increased Bcl-2 and decreased Bax protein and mRNA expression levels, reflecting a decrease in apoptotic cell death in the frontal cortex Anti-apoptotic effects of IH may be in part mediated by the upregulation of *GRP78* and downregulation of *CHOP*, implicating that ERS may play an important role in this process Temperature variability did not affect the anti-apoptotic effects of IH
Vanhuyse et al., 2017^40^	Pig	12	CS induced by coronary ligation	*n* = 6: 38 °C *n* = 6: 34 °C	Lower fluid balance in IH group Lower norepinephrine doses required in IH group Better vascular reactivity in IH group Better dP/dtmax in IH group

N = number; TTM = targeted temperature management; VF = ventricular fibrillation; ECPR = extracorporeal cardiopulmonary resuscitation; ICU = intensive care unit; NDS = Neurological Deficit Score (NDS 0% = normal, NDS 100% = brain death); IH = induced hypothermia; CA = cardiac arrest; NT = normothermia; ABP = arterial blood pressure; CPK = creatine-phosphokinase; ALT = alanine-aminotransferase; NSE = neuron-specific enolase; OPC = overall performance category; CysC = cystatin-C; ECMO = extracorporeal membrane oxygenation; ROSC = return of spontaneous circulation; CPR = cardiopulmonary resuscitation; LV = left ventricle; MRI = magnetic resonance imaging; AST = aspartate-aminotransferase; CS = cardiogenic shock.

In a swine model of CS requiring V-A ECMO support, Vanhuyse et al.^40^ evaluated the effects of mild IH on the cardiovascular system; lower fluid balance and norepinephrine requirements were required in the IH group compared with normothermia. Interestingly, higher vascular reactivity and dP/dt_max_, a marker of left ventricular function, were also observed in the IH group. All these studies have several limitations. First, the number of animals used for these experiments was very limited. Second, protocols applied in these studies were not comparable to the clinical scenario [i.e., healthy animals; short time between the initiation of cardiac arrest and return of spontaneous circulation (ROSC); short follow-up period]. Third, study protocols were all different (i.e., species, depth and duration of hypothermia, speed of rewarming), which limited all further comparisons. Fourth, different temperature ranges were used in the NT groups, according to the studies species, which would limit the translation of these findings to the clinical setting.

## Induced hypothermia in patients undergoing V-V ECMO

Implementation of IH in patients requiring V-V ECMO has been poorly reported. The majority of publications on the use of IH in refractory hypoxemia are case reports describing patients with respiratory failure without ECMO. Three prospective studies described the effects of IH in patients with respiratory failure without V-V ECMO implementation. Despite some limitations (i.e., unknown tidal volume, no standardization of IH protocol), Villar et al.^41^ reported a lower mortality in the IH group (32–35 °C) compared with the standard therapy in a small cohort (*n* = 19) of septic patients with ARDS. Matsuno et al.^42^ demonstrated a lower overall mortality in kidney-transplanted patients admitted to the intensive care unit for acute respiratory failure when treated by IH (i.e., 35 °C) rather than no temperature control. Schorgten et al.^43^ also observed a lower 14-day mortality in septic patients treated with controlled normothermia (36.5–37 °C) compared with no fever control; however, only 50% of these patients had ARDS and no subgroup analysis was realized to assess the effects of such intervention on respiratory function and gas exchanges. Hayek et al.^44^ reviewed all case reports evaluating the benefits of IH in patients with acute respiratory failure, reporting various effects on the avoidance of ECMO and/or improvement in PaO_2_ values.

Table 2 summarizes the studies conducted in patients needing V-V ECMO support for severe ARDS. The first two treated cases of patients with severe ARDS (PaO_2_/FiO_2_ < 100) due to H1N1 infection remained hypoxemic despite V-V ECMO implementation^44^; IH (32–34 °C) was therefore maintained for 24 hours using an external device. Interestingly, for each degree drop in body temperature, mean PaO_2_ concomitantly rose by 7.4 (case 1) and 2.6 (case 2) mmHg, with an average drop in PaCO_2_ of 0.8 (case 1) and 1.5 (case 2) mmHg, respectively. Luc et al.^45^ also reported one patient with severe ARDS secondary to a *Legionella pneumophila* community-acquired pneumonia. Despite the implementation of V-V ECMO, the patient remained severely hypoxemic; IH at 34 °C was then induced for 10 days; and an elevation in PaO_2_ (i.e., from 45 to 75 mmHg) and SaO_2_ (i.e., from 80% to 90%) during the cooling phase was observed and remained consistent over time. Interestingly, Kimmoun et al.^27^ also reported one patient with a refractory hypoxemia secondary to a severe H1N1 infection; by decreasing body temperature from 37 to 34 °C through the heat exchanger of the ECMO circuit, an improvement in SaO_2_, from 82% to 94%, was observed.

**Table 2:** Summary of clinical data evaluating the use of induced hypothermia (IH) during the use of extracorporeal membrane oxygenation (ECMO).

Studies	Design	ECMO configuration	Indication	N	Study protocol	Main outcomes
Hayek et al., 2017^44^	Case report	V-V ECMO	ARDS	2	IH (32–34 °C) for 24 h No information about time to target temperature Device used for IH induction: surface cooling	Correlation between each degree drop in body temperature and PaO_2_ raising and PCO_2_ drop
Luc et al., 2019^45^	Case report	V-V ECMO	ARDS	1	IH (34 °C) for 10 days No information about time to target temperature Device used for IH induction: not mentioned	Correlation between each degree drop in body temperature and PaO_2_ raising
Kimmoun et al., 2013^27^	Case report	V-V ECMO	ARDS	1	IH (34 °C) No information about the period in IH and the time to target temperature Device used for IH induction: heat exchanger connected to the ECMO circuit	Correlation between each degree drop in body temperature and SaO_2_ raising
Levy et al., 2022^50^	RCT Multicentric	V-A ECMO	CS	374	*n* = 168: 33–34 °C for 24 h *n* = 166: 36–37 °C for 24 h IH obtained within 2 h post randomization Device used for IH induction: heat exchanger connected to the ECMO circuit	Non-significant reduction in 30-day mortality rate in IH group (42% vs 51%; *p* = 0.15) Beneficial effects of IH in a composite secondary outcome (i.e., death, heart transplant, escalation to long-term mechanical support or stroke) Similar rate of complications
Beppu et al., 2013^56^	Case report	V-A ECMO	CA - ECPR	1	IH (34 °C) for 24 h IH obtained 3 h after collapse Device used for IH induction: not mentioned	Good neurological outcome
Nusbaum et al., 2014^57^	Case report	V-A ECMO	CA - ECPR	1	IH (32–34 °C) No information about the period in IH and the time to target temperature Device used for IH induction: not mentioned	Good neurological outcome
Moreno et al., 2014^58^	Case report	V-A ECMO	CA - ECPR	1	IH (33 °C) for 24 h No information about time to target temperature Device used for IH induction: heat exchanger connected to the ECMO circuit	Good neurological outcome
Thooft et al., 2014^59^	Case report	V-A ECMO	CA - ECPR	1	IH (33 °C) for 24 h No information about time to target temperature Device used for IH induction: heat exchanger connected to the ECMO circuit	Good neurological outcome
Ikejiri et al., 2021^60^	Case report	V-A ECMO	CA - ECPR	1	IH (34 °C) for 24 h No information about time to target temperature Device used for IH induction: heat exchanger connected to the ECMO circuit	Good neurological outcome
Mita et al., 2017^61^	Case report	V-A ECMO	CA - ECPR	1	IH (34 °C) for 24 h No information about time to target temperature Device used for IH induction: not mentioned	Good neurological outcome
Kim et al., 2017^62^	Case report	V-A ECMO	CA - ECPR	1	IH (34.5 °C) for 96 h No information about time to target temperature Device used for IH induction: surface cooling	Good neurological outcome
Kim et al., 2014^63^	Retrospective Monocentric	V-A ECMO	CA - ECPR	55	No information about the period in IH and the time to target temperature Device used for IH induction: not mentioned	Relation between the application of IH and an increased probability of good neurological outcome in patients with ECPR
Kagawa et al., 2015^64^	Retrospective Multicentric	V-A ECMO	CA - ECPR	87	*n* = 48: <34 °C *n* = 39 ≥ 34 °C Target temperature and durations of cooling/rewarming assigned by the medical physicians regardless of the patients’ conditions Device used for IH induction: cold saline (66%), surface cooling with ethanol evaporation (2%) or with pad (16%), gastric lavage with cold saline (8%), water circulating mattress (60%) patients, cooling device attached to a hemodiafiltration system (9%) patients, heat exchanger with ECMO (28%)	In ECPR subgroup, target temperatures < 34 °C associated with better neurological outcome at 3 months (82 vs 49%, *p* = 0.012)
Pang et al., 2017^65^	Retrospective Monocentric	V-A ECMO	CA - ECPR	79	*n* = 14: 34 °C for 24 h *n* = 65: normothermia for 24 h No information about time to target temperature Device used for IH induction: heat exchanger connected to the ECMO circuit	Better neurological outcome at hospital discharge in patients treated by IH (42.9% vs 15.4%, *p* = 0.02)
Kim et al., 2019^66^	Retrospective Monocentric	V-A ECMO	CA - ECPR	101	*n* = 25: 33–34 °C for 24 h *n* = 76: no control of temperature for 24 h No information about time to target temperature Device used for IH induction: surface cooling	Good neurological outcome in IH group (32%) compared with 34% in the other group (*p* = 0.84) No difference observed in the rate of hospital survival (48 vs 46%; *p* = 0.91) Limitations: o Level of target temperature in the “control” grou*p* = 35.6 °C o Indications for IH subjectively determined by physician and patients’ families
Sakurai et al., 2022^67^	Retrospective Multicentric	V-A ECMO	CA - ECPR	977	*n* = 471: TTM 32 °C (1%) 33 °C (4%) 34 °C (65%) 35 °C (11%) 36 °C (19%) *n* = 506: any control of body temperature Median interval from collapse to reach target temperature: 249′ Median interval of temperature management: 43 hours Device used for IH induction: surface cooling or heat exchanger connected to the ECMO circuit	Better neurological outcome (CPC 1–2 at 1 month after collapse) in ECPR + TTM group (16 vs 10%, OR (95% CI) 1.546 (1.046 – 2.286), p 0.029) In subgroup analysis according to interval from collapse to pump start (>30′, >45′, >60′), better neurological outcome in ECPR + TTM group, except for interval from collapse to pump start > 60′
Nakashima et al., 2022^68^	Retrospective Multicentric	V-A ECMO	CA - ECPR	609	2 sub-groups analysis: ≤ 36 °C for < 12 h (*n* = 136) 34–36 °C for ≥ 12 h (*n* = 250) ≤ 34 °C for ≥ 12 h (*n* = 223)IH duration (≤36 °C) < 12 h (*n* = 136) 12–48 h (*n* = 394) ≥ 48 h (*n* = 79) No information about time to target temperature Device used for IH induction: heat exchanger connected to the ECMO circuit (86%), others (14%)	Significantly lower risk for in-hospital mortality in the 34–36 °C group when compared with >36 °C group (HR 0.73 [0.55–0.96]; *p* = 0.025) Significantly lower risk for in-hospital mortality in the ≤36 °C for 12–48 h group when compared with ≤36 °C for <12 h group (HR 0.69 [0.53–0.90]; *p* = 0.005)
Watanabe et al., 2022^69^	Retrospective Multicentric	V-A ECMO	CA - ECPR	890	*n* = 249: 35–36 °C *n* = 641: 32–34 °C No information about period with TTM Median interval from collapse to reach target temperature: 60′ (NT group) and 110′ (IH group) Device used for IH induction: not mentioned	30-day survival with favorable neurological outcome (CPC 1–2) was similar between the 2 groups (16.5% in NT group, 15.9% in IH group) 30-day TTM complications similar between the 2 groups
Pang et al., 2016^71^	RCT	V-A ECMO	CA - ECPR	21	*n* = 9: 34 °C for 24 h *n* = 12: 37 °C for 24 h No information about time to target temperature Device used for IH induction: heat exchanger connected to the ECMO circuit	Survival rate at discharge with sufficiently good neurological function observed in 2/9 patients in the IH group (34 °C for 24 h) and in 1/12 patients in the NT group (37 °C for 24 h; *p* = 0.37) 6-month mortality and the length of hospital stay similar between groups Limitations: o Number of patients included very small o Patients included in IH group younger o Left ventricular ejection fraction before ECPR lower in the IH group
Chen et al., 2020^72^	Meta-analysis 9 studies	V-A ECMO	CA - ECPR	806	Heterogeneity in TTM protocol applied	Association statistically significative between IH and neurologic outcome / survival in overall
Huang et al., 2022^73^	Meta-analysis 35 studies	V-A ECMO	CA - ECPR	2643	For patients who underwent TTM, goal target temperature ranged from 33 to 36 °C with a median cooling duration of 24 hours across the studies No information about the time to target temperature	No difference in neurologic outcome/survival between TTM and non-TTM group
Duan et al., 2021^74^	Meta-analysis 23 studies	V-A ECMO	CA - ECPR	2035	For patients who underwent TTM, goal target temperature ranged from 33 to 36 °C No information about the period in IH and the time to target temperature	ECPR + IH significantly improved the short-term survival (OR = 2.27, 95% CI (1.60–3.23), *p* < 0.00001) and neurologic outcomes (OR = 2.60, 95% CI (1.92–3.52), *p* < 0.00001) At 3 months of follow-up, ECPR + IH significantly improved survival (OR = 3.36, 95% CI (1.65–6.85), *p* < 0.0008) and favorable neurologic outcomes (OR = 3.02, 95% CI (1.38–6.63), *p* < 0.006)
Bertic et al., 2022^75^	Meta-analysis 92 studies	V-A ECMO	CA - ECPR	6793	TTM applied in 37% of patients No information about the period in IH and the time to target temperature	TTM associated with favorable neurologic outcome assessed by CPC score

N = number; ARDS = acute respiratory distress syndrome; TTM = targeted temperature management; ECPR = extracorporeal cardiopulmonary resuscitation; IH = induced hypothermia; RCT = randomized controlled trial; CA = cardiac arrest; NT = normothermia; V-A ECMO = venoarterial extracorporeal membrane oxygenation; V-V ECMO = venovenous extracorporeal membrane oxygenation; CPR = cardiopulmonary resuscitation; CS = cardiogenic shock; HR = hazard ratio; CPC = cerebral performance category (CPC 1 = good cerebral performance, CPC 2 = moderate cerebral disability, CPC 3 = severe cerebral disability, CPC 4 = coma/vegetative state, CPC 5 = brain death); OR = odd ratio; CI = confidence interval.

To the best of our knowledge, there is no prospective trial evaluating the effects of IH in ARDS patients on V-V ECMO support. Currently, one study is scheduled in France, which will compare the effects of two levels of body temperature (i.e., 33–34 °C vs 36 °C for 48 h) on SaO_2_ in ARDS patients under V-V ECMO (HypoLungECMO, NCT05306392). Whether aiming at NT in these patients would be beneficial in comparison with untreated fever remains unknown.

## Induced hypothermia in patients with V-A ECMO for cardiogenic shock

Many studies assessing IH in CS animal models without V-A ECMO support demonstrated improved physiological parameters, including increased contractility and SV, reduced heart rate and left ventricle end-diastolic pressure, reduced systemic and myocardial oxygen consumption, increased mean arterial pressure, and higher mixed venous oxygen saturation.^46^, ^47^ Systemic biomarkers of ischemia–reperfusion were also reduced with IH in two models of CS.^48^, ^49^

In one randomized trial, Levy et al.^50^ recently studied the effects of IH in patients with CS needing V-A ECMO support. The authors included 374 patients, 168 in the IH group (33–34 °C for 24 h) and 166 in the normothermia group (36–37 °C for 24 h). A trend toward a nonsignificant reduction in 30-day mortality rate was observed in the IH group (42% vs 51%; *p* = 0.15). Interestingly, beneficial effects of IH were also observed in a composite secondary outcome (i.e., death, heart transplant, escalation to long-term mechanical support, or stroke). However, this study had limitations; only patients requiring mechanical ventilation and sedation were included. Moreover, there was consistent heterogeneity in the causes of CS, including a large proportion of patients receiving ECMO after cardiac arrest. Finally, the trial was likely underpowered to statistically detect a survival benefit of 10% with IH. Nevertheless, the use of IH was not associated with more complications (i.e., similar rate of infections and bleeding between groups). The *post hoc* Bayesian analysis also suggested a potential benefit for V-A ECMO in these patients.

## Induced hypothermia in patients undergoing ECPR

European guidelines^6^ currently recommend maintaining a target temperature < 37.7 °C for at least 72 h after ROSC in patients who remain unconscious after cardiac arrest; these recommendations are based on the results of several prospective randomized studies.^51^, ^52^, ^53^, ^54^, ^55^ Currently, there is no recommendation for the use of lower temperature targets in patients with refractory CA needing an ECMO support.

Table 2 summarizes the studies conducted in patients needing V-A ECMO support for refractory cardiac arrest. Most of the published data in this field are case reports or retrospective case series. With the exception of four of them, the time from CA to IH initiation was not reported, while this parameter is the main determinant of cooling effectiveness in this setting.

Few case reports have assessed the effects of IH (33–34 °C) on neurological outcomes in this setting; IH was applied for 24 hours, and the causes of CA were ventricular fibrillation secondary to acute coronary disease or Brugada syndrome,^56^, ^57^, ^58^ Taxus or tricyclic intoxication,^59^, ^60^ pheochromocytoma crisis, and amniotic fluid embolism^61^ or massive pulmonary embolism.^62^ All these cases consistently reported a favorable neurological outcome with cardiac recovery, suggesting the possibility of publication biases.

Kim et al.^63^ retrospectively analyzed a cohort, including 599 patients who experienced an out-of-hospital cardiac arrest (OHCA) in one single center between 2006 and 2013. Using propensity-score matching, the authors observed that the application of IH was associated with an increased probability of good neurological outcome in patients with ECPR. However, this study had some limitations; first, no information was given in the manuscript about the IH protocol that was applied. Second, the criteria for ECPR indication and the upper limit of CPR duration for ECMO implantation were not clearly established. Third, because of a modification in the CPR guidelines during the period of the study, IH was not used before 2010 in patients requiring ECPR.

Kagawa et al.^64^ investigated whether lower target temperatures and/or prolonged cooling could provide improved neurological outcomes in 237 comatose cardiac arrest survivors between 2003 and 2014. Target temperature and durations of cooling/rewarming were assigned by the medical physicians regardless of the patients’ conditions, which would create a selection bias in the analysis of the overall results. However, in the ECPR subgroup, target temperatures < 34 °C was associated with better neurological outcome at 3 months (82% vs 49%, *p* = 0.012) compared with other temperature subgroups. The authors hypothesized that the benefits of ECMO were due to faster cooling and better hemodynamic stability in these patients.

Pang et al.^65^ analyzed retrospectively a cohort of 79 patients requiring ECPR treated between 2003 and 2016; 14 of them received IH (34 °C for 24 h) with a rewarming rate not exceeding 0.5 °C/h after 24 h. Compared with patients with ECPR and normothermia, patients treated by IH had better neurological outcome at hospital discharge (42.9% vs 15.4%, *p* = 0.02). Kim et al.^66^ retrospectively analyzed a cohort including 101 patients who were treated with ECPR, 25 of those receiving IH (33–34 °C for 24 h); at discharge, 32% in the IH group had a good neurological outcome compared with 34% in the other group (*p* = 0.84); moreover, no difference was observed in the rate of hospital survival (48 vs 46%; *p* = 0.91). On the multivariate analysis, the use of IH was not independently associated with neurological outcomes and hospital survival. Importantly, the level of target temperature in the “control” group was 35.6 °C. Indications for IH were also subjectively determined by physician and patients’ families as the use of the active cooling system was not covered by the national health insurance system.

Sakurai et al.^67^ recently performed a retrospective analysis of 977 patients who underwent ECPR for OHCA between 2014 and 2019; the use of TTM (defined in the manuscript as using cooling devices and have any active target temperature strategy) was applied in 471 of them. In ECPR patients undergoing TTM, target temperature was 32 °C (1%), 33 °C (4%), 34 °C (65%), 35 °C (11%), and 36 °C (19%); the median interval from collapse to reach target temperature and interval of temperature management were 249 minutes and 43 hours, respectively. A higher proportion of patients with favorable neurological outcome at 1 month was observed in the ECPR group (16% vs 10%; odds ratio 1.55 [95% CI 1.05–2.29]; *p* = 0.03). These effects were not observed in the subgroup of patients with a time from arrest to ECPR exceeding 60 minutes. Importantly, ECPR patients receiving TTM were younger and had more frequently a cardiac etiology of arrest than others.

Nakashima et al.^68^ recently performed a retrospective analysis of the Extracorporeal Life Support Organization Registry, including 1511 adult patients who underwent ECPR from 2010 to 2019. Of those, 849 patients received IH; the mean duration of temperature ≤ 36 °C was 24 hours, and the mean maximum temperature within 72 hours of ECPR initiation was 36.9 °C. No significant difference in in-hospital mortality at 90 days was observed between the two groups (hazard ratio 1.06 [95% CI 0.93–1.21]; *p* = 0.39). After excluding patients without data on body temperature or who died before IH was completed ended (*n* = 240), IH was associated with a lower probability of in-hospital mortality.

Watanabe et al.^69^ recently analyzed a retrospective cohort of 890 OHCA patients undergoing ECPR between 2014 and 2019; 249 patients were treated with NT (35–36 °C) and 641 with IH (32–34 °C). Whether by using multivariable logistic regression or inverse probability weighting, 30-day survival with favorable neurological outcome (CPC 1–2) was similar between the two groups (16.5% in the NT group and 15.9% in the IH group).

In a prospective observational study, OHCA patients undergoing ECPR had a better neurological outcome than those receiving conventional resuscitation; IH was applied for 24 hours in most of survivors in the ECPR-treated group compared with the control (91.5% vs 54.1%; *p* < 0.0001).^70^ A higher probability of favorable neurological outcome was also observed for a duration of cooling of 24–48 hours and a target temperature of 33 °C compared with other TTM strategies.

One randomized controlled trial^71^ has prospectively evaluated the effects of IH in patients undergoing ECPR. Survival rate at discharge with favorable neurological function was observed in 2/9 patients in the IH group (34 °C for 24 h) and in 1/12 patients in the normothermia group (37 °C for 24 h; *p* = 0.37). Six-month mortality and the length of hospital stay were also similar between the groups. However, this study had some limitations. First, the number of patients included was very small. Moreover, patients included in the IH group were younger, with possibly greater potential for neurological recovery compared with older patients. Left ventricular ejection fraction before ECPR was also lower in the IH group, which might increase the risk of multiple organ failure in these patients.

Four meta-analyses were published evaluating the potential beneficial effects of TH on neurological outcomes after ECPR.^72^, ^73^, ^74^, ^75^ Except for one of them,^73^ all analyses reported an improvement in neurological outcomes for ECPR patients treated with IH. However, the eligible studies and sample size of the included cohorts were relatively limited. Second, most of the studies were observational and retrospective (i.e., very low levels of evidence and high risk of bias) and most of them were unbalanced for baseline characteristics, CA characteristics, and initial neurological status between groups. Third, most of these studies were heterogeneous regarding the inclusion/exclusion criteria for ECPR. Fourth, most of the studies were realized in Asia and could not be generalizable to other settings with other medical standards.

## Complications

Induction of IH is associated with several physiological changes, which could also be harmful in patients requiring ECMO support.^8^, ^76^ Shivering and cutaneous vasoconstriction, ions disturbances (i.e., hypomagnesemia, hypokalemia, hypophosphatemia), cardiovascular and hemodynamic effects (i.e., bradycardia, arrhythmias), coronary vasoconstriction in patients with severely atherosclerotic coronary arteries, mild coagulopathy, unpredictable drugs clearance, risks for infections, and impaired bowel function have been largely described.

In one retrospective cohort, Kagawa et al.^66^ observed a higher rate of arrhythmias and pneumonia in patients with cooling exceeding 28 hours during ECMO than in the others. The rate of bleeding was also significantly higher in patients treated at target temperatures < 34 °C than in others. However, Mecklenburg et al.^77^ recently reported the lack of increased risk for major bleeding in patients needing V-A ECMO for ECPR and treated with IH compared with normothermic ECPR. Similarly, two other retrospective studies^65^, ^66^ also reported IH to be relatively safe during ECPR. Regarding ions disturbances, a retrospective study^78^ including 116 ECPR patients recently yielded that IH was associated with hypokalemia and hypophosphatemia. Shiba et al.^79^ also described an increased risk of pneumonia in ECRP patients treated with IH compared with others. In patients with CS needing V-A ECMO support, IH was also associated with a higher requirement of red blood cell transfusion compared with others, although the percentage of bleeding was not statistically different between groups.^50^

## Conclusions

Severe ARDS, refractory CA, and severe CS are relatively frequent diseases with poor prognosis, which might require the use of V-V or V-A ECMO. Implementation of IH in these patients appears to be a feasible and valuable intervention with some robust rationale and physiological effects. However, the effects on clinical outcome remain uncertain as most of the studies realized in these settings are animal reports, case reports, and retrospective studies. Whether controlled normothermia has an impact on these patients compared with temperature control remains unknown. In this context, further randomized controlled trials are required to better understand the role and impact of TTM/IH in patients requiring ECMO, according to the underlying disease.

## Conflicts of interest

BL received fees for consulting and research grant from Baxter and Amomed. RL and FST received consulting fees from Eurosets. FST received lecture fees from BD and ZOLL.

Conflicts of interest of other authors: none.

## CREDIT authors statement

**AM** and **FST** contributed to the study conception and design. **AM** and **FST** completed data extraction and analysis. **AM** and **FST** drafted the manuscript. **BL**, **FA**, **RL**, **FS**, **MB,** and **FST** revised the manuscript. **All authors** approved the submitted version of the manuscript. **All authors** agreed both to be personally accountable for the author's own contributions and to ensure that questions related to the accuracy or integrity of any part of the work are appropriately investigated, resolved, and the resolution documented in the literature.
